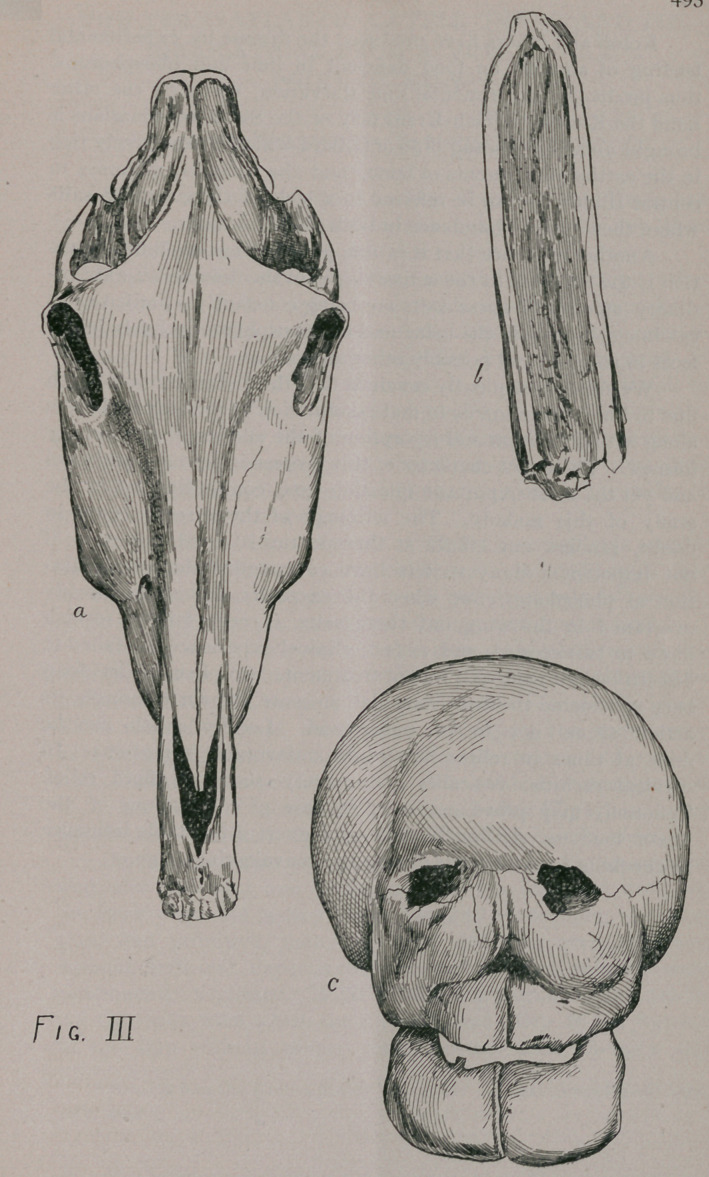# Rachitis*Read before the United States Veterinary Medical Association, September 16, 1891.

**Published:** 1891-10

**Authors:** W. L. Williams


THE JOURNAL

OF

COMPARATIVE MEDICINE AND VETERINARY ARCHIVES.

VOL. XII. OCTOBER, 1891. NO. 10.

### RACHITIS.*

BY W. L. WILLIAMS, V. S.

Rachitis is one of the most widespread maladies to which the animal kingdom is subject, and it may well be doubted if there exists a mammal or bird which is not liable to this disease.

It has been recorded in man, in all our domesticated animals and birds, and in many of the wild animals and birds confined in zoological gardens. So far as we can learn we have no record of the disease among undomesticated and unconfined animals or birds.

The etiology of the disease has so far baffled all investigations, although some of the conditions necessary to the development of the disease have been experimentally demonstrated. It is one of the oldest diseases known to medical literature, having been described by Vegetius.

The malady has received the greatest attention at the hands of writers on human medicine. This has been extremely unfortunate for medical science in general, inasmuch as with this narrowness of the field of observation, errors in our conclusions are apt to creep in, which in a broader area of study would be recognized and eliminated. Still more, we lose by this means of consideration by excluding a large volume of material for study, which is far more available than the disease in man readily offers, either ante or post-mortem.

* Read before the United States Veterinary Medical Association, September 16, 1891.

Much difficulty has been thrown in the way of the study of rachitis in the lower animals by the extremely vague, erroneous and perplexing names under which it has been designated in its various manifestations and by various writers.

In the Mississippi valley and probably over the greater portion of the United States, solipeds offer the most abundant, available and richest field, for the study of the malady, and certainly in no animal have English veterinary writers succeeded in more thoroughly confusing their readers, and in drawing their attention from rachitis, to view under some other more mysterious name this disease under its different forms of manifestation.

One of the chief aims of this paper will be to draw your attention to rachitis as seen in the horse, and thereby enlist your active study in what we believe to be a rich and promising, although sadly abused field. The classification of rachitic diseases has long been a matter of great controversy, and no doubt many of you will dissent to that here proposed.

We find described in man two diseases, which if not identical are certainly very closely allied—rickets or rachitis, and mollities ossium, or osteo-malacia. Trosseau (1) asserts that the two are identical except in age of patient, while Bristowe, (2) although admitting their strong resemblance, believes that the details of anatomical changes are quite different. Without pointing out these differences, however, he recapitulates the symptomatology, morbid anatomy, pathology, course, termination and therapeutics of rickets, and leaves the reader at a total loss as to his reasons for his classification and necessarily the impression that the two descriptions are of one and the same disease, with the difference that rachitis occurs in adults and almost exclusively in child-bearing women.

The difference in the age of the patient seems at present to be the only tangible ground for the differentiation of these in man.

In the lower animals Prof. W. O. Williams, (3) fails to draw any distinction between the various forms of constitutional bone diseases which he describes in such a way as to assist the student in differentiation between them.

Friedberger and Frohner, (4) distinguishes osteo malacia and

---

(1) Theory & Prac. Med., Bristowe, p. 809.

(2) Ibid., pp. 808-809.

(3) Prin. & Prac. Vet. Surgery, pp. 178-199.

(4) Spec. Path. und Therap. Zweite Auflage. Band II. S. 21.

rickets, by the fact that the former is a resoftening of the osseous system of mature animals in consequence of a resorption of lime salts, whereas the latter is a persistence of the soft condition owing to imperfect calcification.

They admit, however, that in many respects they are indentical. They occur simultaneously in the same herd, and experimental research shows that the two diseases are developed in the same manner, and by the same dietetic restrictions, and that the anatomo-pathological differences are those due to a similar if not identical cause acting upon bones which have undergone different stages of development.

It must be admitted by all that rachitis does not consist merely in a want of calcification, but in an actual de-calcification of the bones already considerably developed, so that a one year old foal may have lighter bones than at time of birth; so that both diseases or phases of disease must be due, not alone to a suspension of the calcification process, but to de-calcification of the bone already formed as well.

Prof. Dieckerhoff, fails to give so clear a conception of his idea as to the identity of these two diseases.

We find described quite commonly by veterinary writers several other diseases of domestic animals which if not identical are certainly closely allied to rachitis, such as osteo-porosis, articular rheumatism and partial dislocation of patella.

Friedberger and Frohner, (1) who are certainly correct in their position, classify osteo-porosis with rachitis, as one and the same disease, while Dieckerhoff (2) and Prof. W. O. Williams, (3) dissent and maintain that it is a wholly separate affection. Under the head of partial laxation of the patella, Prof. W. O. Williams (4) describes a disease of young animals attributed by him to grazing on hilly pastures, which in symptoms and course is quite identical with a malady happening not unfrequently in young animals grazing upon the level prairies of the Mississippi Valley.

While, it is certainly undesirable to group together diseases under one head which are essentially dissimilar in their etiology and pathology, it is equally unfortunate to dissociate phases of diseases due to the same fundamental causes, and offering essentially the same characters.

---

(1) Spec. Path. und Therap. Zweit. Auflage.

(2) Lehrbuch. Pathol. u Therap.

(3) Prin. & Prac. Vet. Surg.

(4) Ibid.

In general we would say rachitis is a constitutional disease, affecting principally the osseous system and cartilage characterized usually by an insidious chronic course, with a softening of the bones, increase in size with a marked decrease in the weight of earthly constituents owing to the resorption and excretion of earthy matter, especially of the phosphates and the salts of lime.

The affection occurs mainly in young animals although no age is exempt. The symptoms vary to some extent with the species of animal, the violence of attack, age of animal, etc., beginning usually with a general disorder of the nutritive functions of the body, probably a considerable degree of emaciation, a well-marked lassitude, visibly increased by exercise. In moderately severe cases a well-marked disinclination to move soon becomes apparent, and the patient ceases to join in the play of its companions. In lower animals the temperature is generally slightly, if at all elevated, while in children it appears, there exists a reasonably well-marked fever, with a sensation of heat and with sweating, especially of the head and neck.

There is an increased tendency in all rickety animals to assume the recumbent position, especially well marked in pigs and calves; less evident, however, in horses which, as is well known, naturally maintain the standing posture both in health and disease to a much greater extent than other animals.

Concurrent with or closely following these symptoms are other phenomena of a grave character. The general stiffness, the hobbling gait, becomes more intensified making it next to impossible in many cases for the animal to move, and it will do so only under the goadings of hunger, thirst, or extreme punishment. This stiffness is generally universal over the entire skeleton, but in many cases in the horse, especially those well advanced toward maturity, the cervical region seems to be the chief seat of pathological change.

In many cases this rachitic inflammation of the neck develops quite rapidly, more so than most symptoms of this affection and soon attain an exalted degree. The patient holds the head and neck perfectly rigid as though the vertebræ were all anchylozed, the nose poked out like in poll-evil, the gait extremely stiff and careful, and in turning the animal does so very carefully and without bending any part of the spinal column. Any attempt to forceably bend, raise or lower the head, causes evident pain and the animal perhaps with a groan, attempts to escape punishment by stepping backwards promptly. Some cases in this form are so severe that the animal is unable to get its head to the ground to eat or drink, nor can it reach to any height above its ordinary level to secure hay or other food from a high rack.

In all species of animals and in man there is a well marked tendency to enlargement of the long bones at their distal epiphyses, especially of the radius and tibia, and in man and those lower animals having complete ulnae and fibulae, these are affected simultaneously. These epiphysial enlargements are characterized temporarily by lameness and pain on pressure which in time may subside in one bone to appear elsewhere in another. In horses the lower ends of the cannon bones are frequently involved in this way, and occasionally we find the os suffraginis or os corona affected with a ringbone-like enlargement. In foals one to three years old there is at times noted inflammations of the small bones of the hock, especially of the cuneiform bones, resulting in large bony exostoses (spavins) usually affecting both legs and symmetrical in size and form.

In young foals æt. three to six months, while in good general condition there frequently develops a rachitic hydrathrosis especially of the stifle, more rarely of the tarsal or carpal joints, without in many cases evincing other evidence of disease. Sometimes but one stifle is affected, at other times both, at first usually unassociated with lameness and may so remain throughout its course or gradually developing, the amount of synovia increasing, the fluid pushing the patella forward floating it away from its femoral groove and allowing it to slip over the condyle outwards during the flexion and slipping back in its groove with a snap during extension. In some cases the extensions of the synovial membrane about the peroneus and other muscles passing down over the stifle joint became greatly distended with synovia throughout their entire extent.

This synovial distension with patellar displacement cause the vasti muscles to assume a tense rigid appearance resulting later in atrophy, while the flexor tendons become somewhat contracted, causing knuckling over at the pastern. At the same time, owing to the extra weight thrown upon the well-nigh equally affected front limbs in order to relieve the posterior legs, causes them to bend forward at the knee and pastern joints, resulting in more or less severe contraction of the flexor tendons, occasionally to such a degree that the leg cannot be straightened sufficiently to bring the heel to the ground. The entire bony skeleton suffers in every part from enlargement and rarification, especially noticeable in the epiphyses or the long bones and in the flat bones of the head and face. In the horse this enlargement of the bone is usually best observed in the living animal in that part of the superior maxillary bone bounded by the superior manillary spine, the infra-orbital foramen and the nasal bone, which by enlarging renders the ridge less distinct. Frequently also the thickening of the inferior maxilla is quite distinct. After death the region showing the greatest swelling is usually the anterior part of the superior maxillary bone, which during life is hidden to a great extent from observation by the super-imposed tissues. This enlargement and thickening in the bones of the head and face is quite universal in rachitis in men and animals. The cranial bones are arrested in development in young animals and retain something of the fœtal type, this being specially notable in children and foals.

Nervous derangements have been noted in children, consisting in laryngismus stridulus, convulsions and hydrocephalus. J. Bland Sutton, F. R. C. S., in a highly interesting article on "Rickets" in Monkeys, Bears and Birds, JOURNAL OF COMPARATIVE MEDICINE AND SURGERY, Vol. X. p. 10, relates a case of blindness in a rickety Assyrian bear due to pressure upon the optic nerve by the enlarged surrounding bony tissue. In one foal I observed blindness with apparently some degree of hydrocephalus, and a general want of mental activity was observed.

Deformities of the bony skeleton due to the softened condition of the bones occur in the more advanced stages of the disease and depend largely in form and degree upon the habitual attitude of the patient. In children the deformities are largely those of the spinal column, thorax and lower limbs, and the position of the spinal column being largely upright leads to curvatures in various directions. In the horse the normal position of the body tends to confine spinal displacement mainly to a downward curvature (lordosis) but in rare instances we find an upright curvature (kyphosis) or a lateral curvature (skoliosis) or a union of the two latter (kyphoskoliosis). In pigs and calves the upward spinal curvature usually takes place. Fractures varying in degree are quite prone to occur in almost any part of the skeleton, sometimes complete with marked displacement, at others only partial with bending of the bone (green stick fracture) and may result from struggling when cast or confined in attempts to rise from the recumbent posture in the course of ordinary travel, or what we might term spontaneously from the contraction of muscles.

In the foal these fractures are seen most commonly in the dorsal or lumbar vertebræ during attempts to rise, and result in complete paralysis of the posterior parts necessitating destruction of the animal. Partial fractures of the ribs which occur apparently wholly without accident but due to muscular contraction are quite common and can usually be discovered only by the post-mortem. (See Fig. I, b.) Similar fractures, without apparent cause, occur of various bones, a very good illustration of which we find in (Fig. I, a.)

In work horses fractures of the bones of the legs are likely to occur suddenly while the animal is at ordinary labor, resulting in marked displacement and generally necessitating destruction of the animal. A lack of ossification and even resorption of osseous tissue has been noted in the cranial sutures of children, and we apparently have the same result to a certain degree in (Fig. I, d.,) the case of a foal. The changes in the nasal passage are of such an extent as to cause in some animals a marked difficulty in respiration.

This is recorded as being quite common in pigs and is denominated "Schunffelkrankheit" by the Germans and the same difficulty is not very rare in rachitic foals. Friedberger and Frohner attribute this dyspnœa to thickening of the bones bounding the nasal passages, and this is doubtless largely true especially of the turbinated bones, but in some cases at least the dyspnœa is due rather to a thickening of the nasal septum by deposit between the two cartilagenous laminæ of an abundant soft, spongy tissue, which increases the diameter to such an extent as to largely occupy the nasal passages. This pathological condition is well shown in (Fig. III, b.)

In man and animals dentition is delayed or at times even suspended, and the teeth already erupted become loosened in their sockets, pushed out of their regular positions and may even drop from the mouth. In young patients the growth is generally impeded, emaciation is present in various degrees, and in case of recovery the individual usually retains a more or less dwarfed appearance with short and somewhat crooked legs. When recovery takes place the deformities usually, for the most part remain, rarely interfering with the usefulness of the animal, although much depreciated in value owing to the unsightliness of the deformities. Changes in the form of the pelvis are likely to occur which may render females more or less unable to successfully pass through parturition.

Of the internal organs enlargement of the liver and spleen constitute the chief pathological changes recorded. The bowels are somewhat irregular, and in those animals in which phosphates are normally excreted by the kidneys, we find the urine contains an excess of them, mostly in the form of calcium phosphates, while in solipeds, in which the phosphates do not normally appear in the urine, we find this replaced by a superabundance of calcium carbonate, giving the urine a very thick muddy consistence.

Death from rickets is usually induced by bronchitis, bronchopneumonia, asthenia, or in the lower animals to fractures which demand destruction.

Rachitis in animals is largely confined to certain definite localities or environments. We have noted on three adjoining farms in Central Illinois no less than twenty cases in horses during a practice of twelve years, and outside of this small area during the same time, taken altogether we have not observed an equal number. Other practitioners record similar experiences, thus indicating that there are local causes capable of inducing it, while the occasional appearance of isolated cases indicate that the same or similar conditions exist generally to a minor degree.

After recovery there is in all animals a tendency to hyperossification and hypertrophy of bone, and in some cases cartilages which do not ordinarily ossify, do so quite completely after rachitis.

### Cases in Practice.

*A.* An unusually large, heavy-boned muscular, vigorous, full-blood Norman colt, age at present 3½ years. He was unusually robust up to about four months of age, when a synovial distension of one stifle appeared, soon followed by similar changes in the other leg. The enlargements slowly increased, lameness in both stifles supervening within a few weeks, coupled with some degree of emaciation and lassitude. The lameness gradually increased in severity until eight or ten weeks after the inception of the malady he could not rise without assistance, but once upon his feet could manage to walk about the stall with difficulty, being quite rigid in his limbs and back. For three months the owner assisted him to his feet two or three times a day. Emaciation advanced rapidly during this time, but the growth of the foal suffered but little interference. The patellæ were soon floated out beyond their groove so that they could readily pass out and in over the external condyles of the femur. The distended synovial sacs were tense, somewhat hot and tender. The relaxation of patellar ligaments permitting luxation of patellæ, with accompanying contraction of the vasti muscles, caused the stifle joints to be held in a peculiar extreme extension, with flexion of the hock and very distinct flexion and knuckling of the fetlocks. The anterior limbs suffered similarly as the disease advanced, and the elbow joint became the seat of similar changes observed in the stifles, while the flexor tendons contracted to such a degree that the foal walked on the anterior part of fetlock joint.

The appetite was good, temperature about normal, bowels somewhat constipated. At about the ninth or tenth month of age, or the fifth or sixth month of disease, the symptoms slowly abated, the colt gradually regained flesh and spirits, the synovial distension of stifles declined, while the ends of some of the long bones apparently thickened, and the bones of the head and face became distinctly enlarged. The contractions in the flexor tendons of the fore-legs persisted, and at about fourteen months tenotomy was performed. From that time he has gradually improved, and is now a horse of about 1750 lbs. weight, which is probably 300 lbs. less than he would have attained barring accident.

He now has every appearance of health and vigor, has served mares readily and probably successfully, is reasonably active in his movements, although showing some degree of awkwardness and immobility; his stifles are still extended, the vasti muscles apparently stretched and the patellæ still occasionally slip to and fro over the external condyle. There is some bending forward of the carpal joint and apparently an increase in the normal curvature of the radius, probably also of the metacarpus.

Some two years prior to the affection in this colt, a highly bred trotting mare, aged at the date of disease four years, property of same owner and kept on same premises as the above, developed extreme lardosis or sway-back, within a very few months, without revealing other symptoms of disease except slight stiffness of gait and a general lassitude. She made a prompt recovery, the deformity persisting.

In the year following that in which the colt became affected, there occurred on the same farm no less than four cases of synovial distension of the stifle in sucking foals, some becoming quite lame, one or two suffering from emaciation, and in several there was partial dislocation of patellæ. Three of them have recovered, the other has markedly improved.

During the following season in 1890, one mild case of synovial distension at stifle appeared, which has, it seems, recovered. At present one of the colts of the year's crop in which four cases of synovial distension of stifle occurred, but not included in the four affected, he having always appeared in the best of health, has recently developed a tendency to hypertrophy of the splint bones, especially of those of the fore-legs, which now extend down to the enlargement at the extremity of the metacarpal bone, and are nearly one inch in thickness, giving the fore limbs a very peculiar aspect. The animal is in perfect health apparently, and moves naturally.

Recently a four year grade draft mare, in the same herd, has become extremely stiff and sore in her neck, walks stiffly as though in pain, maintains the entire spinal column in a straight line, neck especially held quite rigid with nose poked out. Temperature slightly elevated (102 F.), pulse normal, appetite good, emaciation slight. Any attempt to forcibly raise, lower or bend head or neck to right or left, causes great pain, and the animal starts backwards to escape punishment. Enlargement of the facial bones is progressing slowly. The urine from this mare, and several of the colts taken during the active stages of the disease, show uniformly a large excess of calcium carbonate.

This group of cases on the same farm, and under the same management, demonstrates to an excellent degree, the variations which this disease may assume. The first case serves as a connecting link between the rather common, mild rachitic synovial distension of the stifle joints in young foals, and the enlargement of the facial bones known to English veterinary writers as osteoporosis. The enlargement of the facial bones in this animal is now so evident, that no one could avoid so classifying it.

*B*. A full-blood French draft foal, age about eight months; location the adjoining farm to *a*. When first observed the foal was down and unable to rise, assisted to its feet, it was unable to stand. There was complete paralysis of posterior parts with the appearance of broken back. The foal was destroyed, and an autopsy revealed a transverse fracture of one of the lumbar vertebræ with dislocation and pressure upon the spinal cord. The bones throughout the entire system were soft, porous and easily cut with a knife. Prior to my visit two or three foals of same age had succumbed in the same manner, doubtless from fracture, and several more died after my visit.

These weanling foals were all kept in a warm basement not greatly crowded, and were highly fed. All were unthrifty, emaciated, lame in various joints, stiff, some with arched backs, others the opposite. The disease was not confined at this date to weanling foals, but affected as well yearlings and two-year-olds kept in another part of the stable. One two-year-old colt exhibited the disease mainly in his hocks in the form of large bone spavins, causing considerable lameness and not yielding to treatment, while others of the same age showed principally the enlargement of the facial bones. Some seven or eight out of about twelve of the year's colt crop succumbed to the disease, the others recovered with so much deformity and interference with development, that they were of little value for breeding purposes for which they were designed. Of the older colts some four or five yearlings and two year olds became affected with, as nearly as remembered, one death, the others recovered with persistent deformities, which largely diminished their value. The disease had been seen on the farm before, but there had been a lapse for several years. Two or three years prior to our date of observations, we saw on the same farm a yearling colt with supro-lateral curvature of the spine (Kyphoskoliosis), which made a good recovery with slight deformity and sold at good price for breeding purposes. Subsequent to the time above mentioned, about 1885, no cases have been observed on this farm.

*C.* An eight months common filly, of light breed, on a farm adjoining that on which the group marked *a* was kept. She appeared in her usual health until about three months old, when she ceased to grow, seemed unthrifty, would not join in the play and gambols of its associates. Its movements were slow, careful and indicative of weakness. Within a few weeks extreme lordosis was developed so that the middle of the back had sunken downwards fully five inches. When about eight months old the animal was procured for special study, and was led with difficulty about two miles, arriving in an exhausted condition. The temperature was slightly elevated, the appetite fair, the bowels somewhat constipated, urine scant and thick, and contained an enormous excess of calcium carbonate. There was extreme emaciation, with enlargement of the abdomen. The respirations were hurried and difficult, the dyspnœa being in part nasal, largely pulmonary. A white froth escaped from the nostrils in considerable amount. The lungs were somewhat dull on percussion throughout, especially marked at their interior border. Auscultation revealed slight broncho-pneumonia. The exertion required of the foal in traveling from the farm to the infirmary seemed to act unfavorably upon the course of the disease, and the patient rapidly grew worse, the pulmonary symptoms becoming aggravated until in about one week, it was found dead one morning upon entering the stall. The extreme lordosis so evident during life was not distinguishable after death—the relaxation of the muscles and relief from weight had caused the spinal column to assume its normal posture.

Autopsy revealed in the main: First, a well defined thickening of the flat bones, especially of the face (Fig. I, d), and inf. maxilla (Fig. II, a), pathological phenomena which thoroughly identified it in this respect with osteo-porosis.

Second, curvature of bones as shown in one of the asternal ribs, (Fig. I, c,) antero-posterior curvature and (II, c,) a minor degree of lateral curvature of the same rib, irregular toward the head and an anterior-posterior curvature of the radius beyond that observed in health.

Third, Fractures of an apparently spontaneous kind and incomplete character (green stick) of three ribs. Two asternal on opposite sides, one sternal as shown (in Fig. I, b), and also a fracture of the left ramus of inf. maxilla, as seen in (Fig. II, a.) This bone also shows slight lateral curvature.

Fourth, an extremely light, porous fragile condition of the entire bony skeleton.

Fifth, enlargement of the liver.

This case illustrates, at one and the same time, with equal strength, rickets and osteo-porosis.

*D*. A well-bred trotting filly, age, at beginning of observation, 2½ years, when she exhibited a marked stiffness and soreness of the neck, so that she could with difficulty lower her head sufficiently to graze. The neck could not be bent sideways nor the head elevated beyond the straight line without great pain to the animal.

Repeated severe blisters over the course of the cervical vertebræ caused a slow subsidence of the symptoms, which however, never fully disappeared.

Some months later, in addition to progressive emaciation, the animal exhibited signs of dyspnœa, when she began to gain in flesh and spirits.

An examination of the nostrils showed the cause to be mainly a thickening of the septum nasi, and after a brief wait succeeding

Fig. I

tracheotomy, the septum, (Fig. III b), was removed as high as practicable.

It was found to be almost one inch in thickness in the lower part of the nostril, the two layers of cartilage having been pushed apart by a spongy new formation. Higher up in its extent the cartilages approached each other, and the septum resumed about its usual thickness. Within a few days the tracheotomy tube was removed, and the case seemed to progress rather favorably for a time, but after several months the dyspœnea returned and tracheotomy was again called to our aid, when we found to our surprise, that at the seat of our former operation, the trachea was thoroughly ossified for several inches and the tube much narrowed, necessitating a lower operation, which afforded but little relief, and the animal succumbed a few weeks later. Late in the disease, thickening of the facial bones had progressed to a decernable degree, and when after death, prepared for preservation, it exhibited the appearance delineated in (Fig. II, b.) Autopsy further revealed old fractures of two or three ribs.

*E.* A well-bred roadster colt, age now, three and one-half years. He is very large, muscular and well developed, and has at no time been debilitated nor emaciated, neither has he exhibited any lassitude or disinclination to play with his associates. He became lame when about three months old and exhibited hard osseous tumors at the lower epiphyses of the metatarsals and metacarpals, first appearing in one leg, remaining a few months, disappearing slowly and imperfectly only to re-appear in another. Later, the lower epiphyses of radii and tibiæ, suffered in a like manner, giving rise to considerably sized epiphysial enlargements, which, like those of the cannon bones have slowly disappeared. The last appearance of lameness was in one fore foot, where it has persisted now for more than a year.

This series of cases demonstrate reasonably well the symptoms and progress of rachitis in the horse and we believe serves to connect into one group, in a tangible manner, the varied phenomena observed in different individuals, and which, by various writers, have been separated into different affections.

In order to facilitate comparison to a certain degree between rickets in man and the horse, the celebrated Jadelot cranium (*) is here sketched (Fig. III, c.) which although an extreme specimen, bears a striking ressemblance in many essential points to the rachitic facial and cranial bones often see in our patients.

* Journal Comparative Medicine and Surgery, Vol. X, 1889.

FIG. II

Fig. III, a. represents the healthy head of the horse.

The differential diagnosis of rachitis in the horse is comparatively easy. It may be confounded: First, in quite young animals with pyo-septhaemia or omphalo-plebitis of new-born foals, which usually has a more or less clear history, develops usually within a few days or weeks after birth, usually shows some slight indication or infection at the umbilicus, the joint complications are more sudden and severe and the epiphises of the long bones are not affected.

Second, in animals of diverse age, accompanied by lameness or stiffness of neck, with articular rheumatism which is more sudden in its outset than rachitis, more prone to metastasis and affects the joint, not the epiphyses.

Third, in cases of facial enlargement, with odotomes, or other dental affections, which, unlike rachitis, are seldom symmetrical and rarely show in that part of the face most prone to rachitic change, are usually sharply defined in outline and, except respiration or mastication is interrupted, they rarely affect the general condition of the animal unfavorably.

Fourth, in those cases of cervical rachitis, with cervical articular glanders, which is more sudden and severe in its outset and usually exhibits laryngeal or pulmonary symptoms (cough) or farcy.

The etiology of rachitis is not yet definitely determined. Climate, altitude, geological formation, species, breed, food and and housing seem incompetent to prevent it as sporadic cases appear everywhere. And yet there is something about food and environment which makes disease common in one locality rare in others. It has not been recorded in free wild animals and birds while those confined in zoological gardens suffer very seriously from its ravages. In the crowded tenements of London it proves a veritable scourge among the children of the poor and yet it appears although far less rarely, in the homes of the wealthy and under the best known sanitary regulations. It seems also that in those localities where rickets prevails, that it confines its ravages mainly to one species of animals. In one locality it apparently affects mainly cattle, in another, pigs, in another, colts. The character of the available litterature gives no clue to the grounds for this apparently peculiar condition. Possibly it may be due to the fact that the disease attacks mostly that species of animal to which most attention it paid in a given locality and hence a higher state of domestication and more confining environments.

FIG. III

Roloff and others have produced the disease by experimental feeding of animals on food deficient in lime and phosphorous, thus producing experimental lime starvation, while on the other hand the Kleie or Hirsch-Krankheit of the Swiss veterinarians is brought about by feeding bran or pollard which is excessively rich in the earthy constituents of bone, and the rachitis of horses in central Illinois cannot be referred to a lack of these earthy salts where they get an abundance in both food and water.

Another evidence that it is not phosphorous or lime starvation is the fact that in the active stages of the disease an extraordinary amount of these salts are being constantly eliminated as calcium carbonate in the urine and the phosphates probably in the feces in solipeds and possibly other animals.

We must consequently conclude that the disease is not so much due to lime starvation as to mal-assimilation of the ingested salts along with a solution and resorption of the osseous tissue already formed. The exact character of this process is the most stubborn and yet the most important question now confronting us in the study of this malady. The ætiology of the disease being in doubt, renders our efforts at therapeutics at least uncertain, if not impotent. Many writers have commended the administration of phosphates, but when the excretory organs are already overtaxed in throwing out these salts, it seems folly to furnish more to be excreted, and in fact, clinical experience has failed to discern benefit from this line of treatment. Cod liver oil has long been considered the best remedial measure known although its action seems not to be understood. Locally much can be done at times to relieve urgent or unfavorable symptoms. In cervical rachitis, vesicants and cautery seem to afford relief. Tenotomy may prove necessary in case of contractions of the flexor tendons. Iodine blisters may exert a favorable influence in checking bony deposits during the convalescing stage.

## Figures and Tables

**Fig. I f1:**
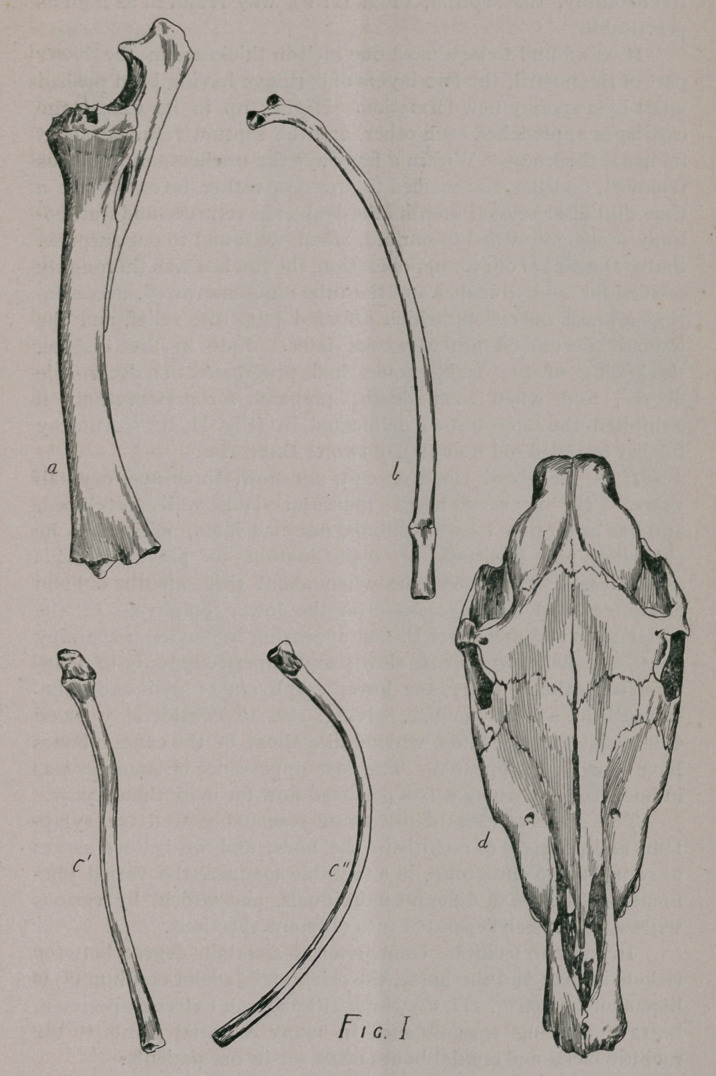


**Fig. II f2:**
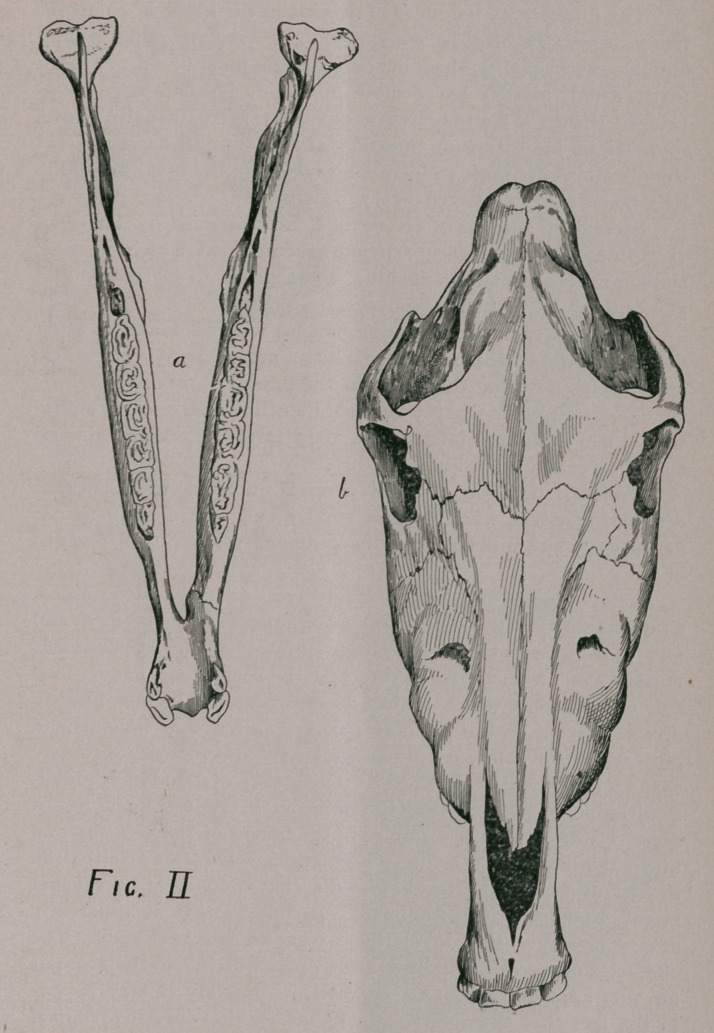


**Fig. III f3:**